# Birth size after embryo cryopreservation: larger by all measures?

**DOI:** 10.1093/humrep/dead094

**Published:** 2023-05-13

**Authors:** E Landsverk, K Westvik-Johari, L B Romundstad, S Opdahl

**Affiliations:** Department of Public Health and Nursing, Faculty of Medicine and Health Science, Norwegian University of Science and Technology, Trondheim, Norway; Department of Public Health and Nursing, Faculty of Medicine and Health Science, Norwegian University of Science and Technology, Trondheim, Norway; Division of Obstetrics and Gynecology, Department of Fertility, St. Olavs Hospital, Trondheim University Hospital, Trondheim, Norway; Centre for Fertility and Health, Norwegian Institute of Public Health, Oslo, Norway; Department of Public Health and Nursing, Faculty of Medicine and Health Science, Norwegian University of Science and Technology, Trondheim, Norway

**Keywords:** birth size, ponderal index, head circumference at birth, placental weight, birthweight:placental weight ratio, frozen embryo transfer, fresh embryo transfer, assisted reproduction, registry-based study, sibship study

## Abstract

**STUDY QUESTION:**

Are the changes in birthweight after frozen and fresh embryo transfer associated with corresponding changes in other measures of foetal growth and placental efficiency?

**SUMMARY ANSWER:**

Although placental efficiency was reduced for both frozen and fresh embryo transfer, children born after frozen embryo transfer (frozen-ET) had symmetrically increased size at birth, whereas children born after fresh embryo transfer (fresh-ET) were asymmetrically smaller at birth, compared to naturally conceived children.

**WHAT IS KNOWN ALREADY:**

In pregnancies following frozen-ET, the risk of being born large, as measured by birthweight, is higher than after natural and fresh-ET conceptions. It is not known whether this is a result of symmetrically increased growth and increased placental efficiency.

**STUDY DESIGN, SIZE, DURATION:**

A Norwegian nationwide registry-based cohort study of 3093 singletons born after frozen-ET, 15 510 singletons born after fresh-ET and 1 125 366 singletons born after natural conception from 1988 to 2015 was performed. We identified 6334 sibships with at least two different conception methods.

**PARTICIPANTS/MATERIALS, SETTING, METHODS:**

Data were collected from the Medical Birth Registry of Norway and the Norwegian National Education Database. Main outcome measures were birth length, birthweight, head circumference, ponderal index (birthweight relative to birth length in kg/m^3^), placental weight, birthweight:placental weight ratio, gestational age, and birthweight *z*-score. We estimated mean differences between children born after frozen-ET and fresh-ET compared to natural conception, at the population level and within sibships. Adjustments were made for birth year, maternal age, parity, and education.

**MAIN RESULTS AND THE ROLE OF CHANCE:**

Estimates at the population level and within sibships were consistent for all outcomes, for both fresh and frozen-ET compared to natural conception. Within sibships, children born after frozen-ET had longer mean length (*Δ* = 0.42 cm, 95% CI 0.29 to 0.55) and head circumference (*Δ* = 0.32 cm, 95% CI 0.23 to 0.41) at birth, but a similar ponderal index (*Δ* = 0.11 kg/m^3^, 95% CI −0.04 to 0.26), compared to naturally conceived. Children born after fresh-ET had a shorter length (*Δ* = −0.22 cm, 95% CI −0.29 to −0.15) and head circumference (*Δ* = −0.15 cm, 95% CI −0.19 to −0.10), and lower ponderal index (*Δ* = −0.15 kg/m^3^, 95% CI −0.23 to −0.07) at birth compared to natural conception within sibships. Furthermore, mean placental weight was larger after both frozen-ET (*Δ* = 37 g, 95% CI 28 to 45) and fresh-ET (*Δ* = 7 g, 95% CI 2 to 13) compared to natural conception within sibships, whereas mean birthweight:placental weight ratio was reduced for both frozen-ET (*Δ* = −0.11, 95% CI −0.17 to −0.05) and fresh-ET (*Δ* = −0.13, 95% CI −0.16 to −0.09). A range of sensitivity analyses all gave similar conclusions as the main models, including restriction to full siblings, restriction to single embryo transfer, and adjustment for maternal BMI, height, and smoking.

**LIMITATIONS, REASONS FOR CAUTION:**

Additional adjustment for maternal BMI, height, and smoking was possible only for a small sample of the study population (15%). Data on causes and duration of infertility, as well as treatment details, were limited.

**WIDER IMPLICATIONS OF THE FINDINGS:**

The increased birthweight observed in singletons after frozen-ET is associated with a symmetrically increased birth size and large placentas, also after controlling for maternal factors through sibship analyses. Identifying the responsible treatment factors and the long-term health outcomes are particularly important considering the increase in elective freezing of all embryos.

**STUDY FUNDING/COMPETING INTEREST(S):**

This work was partly supported by the Central Norway Regional Health Authorities (project number 46045000), the Norwegian University of Science and Technology (project number 81850092) and the Research Council of Norway through its Centres of Excellence funding scheme (project number 262700). The authors have no conflicts of interest to declare.

**TRIAL REGISTRATION NUMBER:**

N/A.

## Introduction

Children born after ART comprise an increasing proportion of birth cohorts in Europe (Wyns *et al.*, 2022). In Norway alone, more than 3000 children are born following ART-treatment annually (Norwegian Institute of Public Health, 2022). Over 30% of all children born after ART in Europe originate from frozen embryo transfer (frozen-ET) (Wyns *et al.*, 2022). In pregnancies following frozen-ET, the risk of hypertensive disorders in pregnancy (HDP) and of being born large for gestational age (LGA) are higher, and the risk of being born small for gestational age is lower, than in ART-conceived pregnancies with fresh embryo transfer (fresh-ET) (Luke *et al.*, 2017; Berntsen and Pinborg, 2018; Maheshwari *et al.*, 2018; Elias *et al.*, 2020). These findings have been confirmed in three recent sibship studies with natural conception (NC) as the reference group (Purkayastha *et al.*, 2021; Westvik-Johari *et al.*, 2021; Petersen *et al.*, 2022).

LGA can result from symmetric or asymmetric growth, defined by ponderal index at birth (Lepercq *et al.*, 1999), which is a measure of birthweight relative to birth length. It has been proposed that a symmetrically large size at birth reflects constitutional and genetic factors, whereas an asymmetrically large size reflects an abnormal intrauterine metabolic environment (Van Assche, 1997). Indeed, the asymmetric growth pattern may be more prevalent in pregnancies among diabetic women (Persson *et al.*, 2011). Whether the increased foetal growth in frozen-ET is symmetrical or asymmetrical is largely unknown. Such knowledge could give insight into the underlying mechanism of their high birthweight.

One distinct difference between fresh-ET and frozen-ET cycles is the supraphysiological hormone levels following controlled ovarian stimulation (COS) at the time of embryo transfer in fresh-ET (Manna *et al.*, 2022). COS has been shown to advance development of the endometrium leading to embryo-endometrium asynchrony at the time of implantation in fresh-ET (Kalakota *et al.*, 2022), but whether this further impairs placentation and placental efficiency is unclear (Manna *et al.*, 2022). Placental function is of critical importance for foetal growth, and the birthweight:placental weight ratio can be viewed as a measure of placental efficiency, as it directly translates to grams of foetus per gram placenta. This ratio is lower in ART compared to naturally conceived singleton pregnancies (Haavaldsen *et al.*, 2012), but it is not known whether it differs between frozen-ET and fresh-ET.

The primary aim of this study was to investigate whether the now well-documented increased birthweight in frozen-ET is a result of symmetrical or asymmetrical foetal growth, and whether it is accompanied by increased placental efficiency.

We used comparisons within sibships to control for confounding from unmeasured and unknown parental factors, such as genetics, preconception lifestyle, and health (Lawlor *et al.*, 2016; Frisell, 2021).

## Materials and methods

### Data sources

Data were obtained from the Medical Birth Registry of Norway (MBRN), which was established in 1967, and collects information on all deliveries in Norway through a standardized notification form, completed by midwifes or doctors shortly after delivery. The MBRN collects information on maternal health before and during pregnancy, complications during pregnancy and delivery, as well as data on perinatal health. Ever since the first baby was born after IVF in Norway in 1984, MBRN has also collected information on ART treatment cycles resulting in ongoing pregnancies, directly from all ART clinics in Norway. The ART notification form contains information on mode of fertilization (ICSI/IVF), culture duration, and number of embryos transferred. Since 1988, information on embryo cryopreservation has also been recorded. After disclosure from the mother, midwifes also register ART-conception on the birth notification form, but details from the treatment will be missing. Following a revision of the delivery notification form in December 1998, information on maternal smoking and placental weight was included. Maternal height and weight have been recorded since 2007, but with a substantial proportion of missing data in the first years. The unique national identity number of each Norwegian resident was used to link information from MBRN to yearly information on education for women who gave birth during the study period, as recorded in the Norwegian National Education Database at Statistics Norway.

### Study factors

ART treatment was defined as any fertilization outside the female body, and exposures were defined as subsequent transfer of fresh or frozen embryos. Outcomes were the following measures of birth size: birth length, ponderal index (birthweight relative to birth length in kg/m^3^), head circumference at birth, placental weight, birthweight:placental weight ratio, and corresponding *z*-scores. To facilitate comparison with previous studies (Purkayastha *et al.*, 2021; Westvik-Johari *et al.*, 2021), we also included gestational age, birthweight and birthweight *z*-scores as outcomes. Placental weight was measured shortly after delivery with umbilical cord and membranes attached (Staff *et al.*, 2020), while birth length and head circumference were rounded to the nearest centimetre using standard measuring protocols. Pregnancies without registration of ART treatment were considered as naturally conceived and included non-ART fertility treatments such as insemination and ovulation induction with natural fertilization. For NCs, gestational age was estimated by routine ultrasound examination performed in Week 18–20 of pregnancy. If this information was missing, the date of last menstrual period was used. For ART-conceived pregnancies, gestational age was based on Week 18–20 ultrasound screening and if this information was missing the date of embryo transfer was used. We used Marsal’s formulas based on intrauterine measurements (Marsal *et al.*, 1996) to calculate birthweight *z*-scores according to gestational age and sex, defining one SD as 11%. In addition, we calculated *z*-scores for all birth size measures using measured values at birth after NC as the standard, with fourth-order fractional polynomial regression to estimate expected birth size and SD according to gestational age in days and sex (Ohuma and Altman, 2019).

### Study population

**Figure 1.**
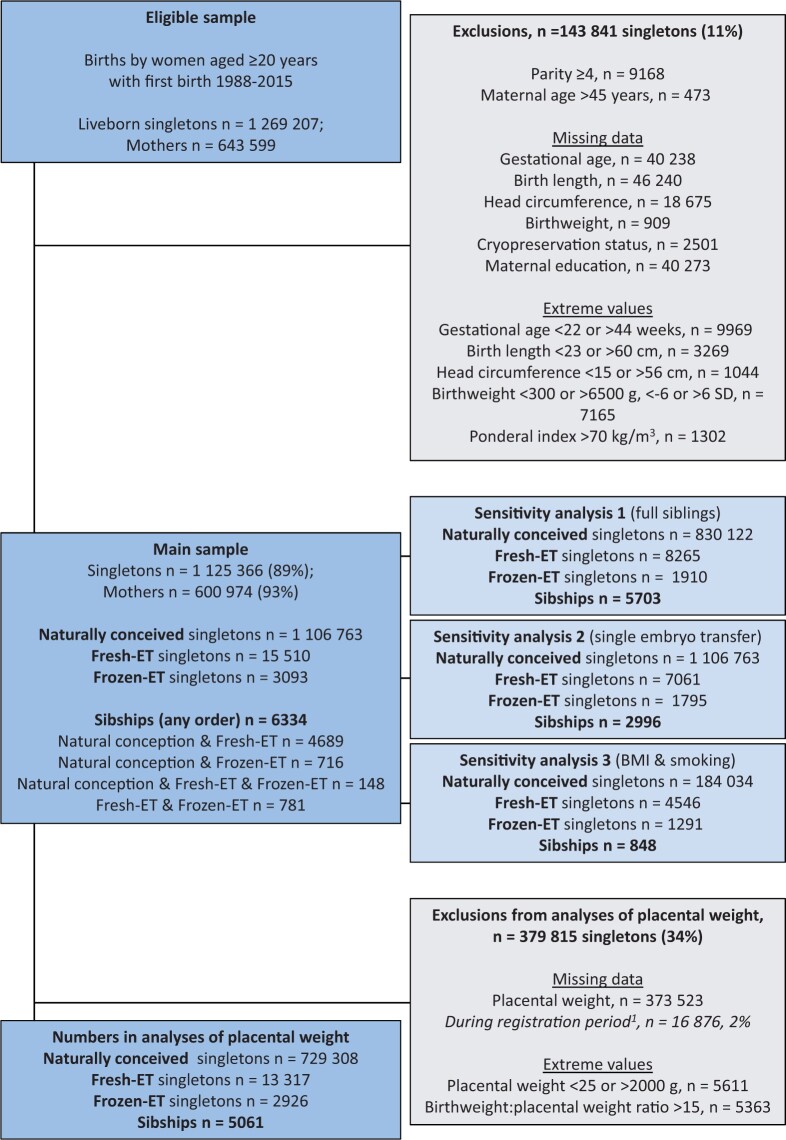
**Flow chart of study population showing eligibility, exclusion criteria and numbers, and number of exposed and unexposed observations in the analyses’ samples.** ^1^Placental weight was recorded from December 1998. If not otherwise specified, sibships refer to sibships with at least two of the three different conception methods. Fresh-ET, fresh embryo transfer; frozen-ET, frozen embryo transfer.

The study population was defined similarly as previous sibship studies (Westvik-Johari *et al.*, 2021; Petersen *et al.*, 2022). We defined our study period from 1988, when the first child from frozen-ET was born, until 2015. Eligibility was defined as liveborn singletons whose mothers delivered their first child within the study period and at age ≥20 years (1 269 207 infants with 643 599 mothers, Fig. 1). Deliveries up to the first four by each mother were included (Parity 0–3). We excluded deliveries at maternal age >45 years. These criteria ensured comparable range of maternal age and parity between ART and NCs while maximizing the number of sibships in the analysis sample. We further excluded ART-conceived singletons with unknown cryopreservation status, and singletons with unknown outcome data or maternal education, as well as singletons with extreme values or implausible combinations of gestational age and birth size (Fig. 1). After these exclusions, our study population (main sample) comprised 1 125 366 infants with 600 974 mothers, where 15 510 were born after fresh-ET and 3093 after frozen-ET. In this sample, there were 6334 sibships with at least two of the three different conception methods. We further excluded extreme or missing values of placental weight and birthweight:placental weight ratio, which resulted in 745 551 infants, and 5061 sibships with at least two of the three different conception methods, with data on placental weight.

### Statistical analysis

We calculated mean differences with 95% CIs for ART versus NC at the population level and within sibships, using multilevel linear regression models with deliveries as one level and mothers as another (i.e. one sibship per mother). Population level associations were estimated in random intercept models. Associations within sibships were estimated in models with a fixed intercept for each woman (also referred to as a ‘conditional’ model) (Rabe-Hesketh and Skrondal, 2012). In the conditional model, only differences within sibships contribute to the estimate. Potential confounders were factors which could influence our outcomes and the need for ART treatment. We included the following as categorical covariates: year of birth (88–95, 96–97, 98–99, 00–01, 02–03, 04–05, 06–07, 08–09, 10–11, 12–13, 14–15), maternal age (20–29, 30–34, 35–37, 38–40, 41–45 years), parity (0, 1, 2, 3), and maternal education in the year of delivery (high school or lower, college/university lower degree, college/university higher degree).

We investigated the robustness of our data by restricting the main sample to relevant subpopulations. First, we restricted to mothers with two or more singletons with the same father (full siblings), to control for confounding by constant paternal factors such as ethnicity and height. Second, we restricted ART-pregnancies to those conceived after single embryo transfer. Third, we restricted analyses to only include deliveries with complete data on maternal height, pre-pregnancy BMI, and smoking (15% of main sample), and additionally adjusted for maternal height as a continuous covariate, BMI as a categorical covariate (<18.5, 18.5–24, 25–29, 30–34, ≥35 kg/m^2^), and any smoking during pregnancy as a categorical covariate (yes/no). Estimates within sibships were not adjusted for maternal height, which is constant within sibships.

To investigate whether associations were driven by specific combinations of parity and conception method, we restricted the main sample to mothers’ first and second delivery and used random intercept model analyses with an interaction term between parity and a categorical covariate containing all possible combinations of conception method for the first and second delivery (i.e. NC–NC, NC–fresh-ET, NC–frozen-ET, fresh-ET–NC, fresh-ET–fresh-ET, fresh-ET–frozen-ET, frozen-ET–NC, frozen-ET–fresh-ET, frozen-ET–frozen-ET). Further, we adjusted for birth year, maternal age, and education, and obtained predicted means for each outcome.

We evaluated the normality assumption of residuals using QQ-plots, density distribution plots and histograms. Assumptions were met for all outcomes.

All statistical analyses were conducted using Stata Statistical Software, version 17 (StataCorp LP, College Station, TX, USA).

### Ethics

The study was approved by the Regional Committees for Medical and Health Research Ethics (REK 2010/1909).

## Results

### Description of the study population

Table 1 describes the study population and shows that the number of children born after ART-conception increased over the study period, and a large increase in the number of children born after frozen-ET was seen from year 2002. On average, women who gave birth after ART were older, had lower parity, smoked less, and had higher education than women who gave birth after NC. Among singletons born after frozen-ET, 39.8% were conceived using ICSI, and 58.0% were born after single embryo transfer. Among singletons born after fresh-ET, a similar proportion as in frozen-ET were conceived using ICSI (39.9%), whereas single embryo transfer was less common (45.5%).

**Table 1.** Description of the study population, 1 125 366 liveborn singletons in Norway 1988–2015, according to conception method.

	Natural conception	ART with fresh-ET	ART with frozen-ET
Number of observations, n (%)	1 106 763 (98.3)	15 510 (1.4)	3093 (0.3)
Birth year, n (%)			
1988–1996	276 183 (25.0)	1305 (8.4)	72 (2.3)
1997–2001	211 623 (19.1)	1699 (11.0)	99 (3.2)
2002–2006	213 234 (19.3)	3238 (20.9)	339 (11.0)
2007**–**2011	227 908 (20.6)	4944 (31.9)	1259 (40.7)
2012**–**2015	177 815 (16.1)	4324 (27.9)	1324 (42.8)
Mean maternal age, years (SD)	29.4 (4.8)	33.5 (4.1)	33.5 (4.1)
Parity, n (%)			
0	541 878 (49.0)	10 895 (70.2)	1619 (52.3)
1	395 970 (35.8)	3935 (25.4)	1210 (39.1)
2	140 750 (12.7)	569 (3.7)	228 (7.4)
3	28 165 (2.5)	111 (0.7)	36 (1.2)
Maternal education, n (%)			
High school or lower	607 896 (54.9)	6434 (41.5)	1203 (38.9)
College/university lower level	394 868 (35.7)	6685 (43.1)	1391 (45.0)
College/university higher level	103 999 (9.4)	2391 (15.4)	499 (16.1)
Maternal smoking in pregnancy^1^, n (%)			
Non-smoker	548 800 (72.9)	11 127 (81.5)	2574 (86.1)
Smoker^2^	92 127 (12.2)	695 (5.1)	112 (3.7)
No consent to registration^3^	100 444 (13.3)	1652 (12.1)	279 (9.3)
Missing^4^	11 665 (1.5)	179 (1.3)	23 (0.8)
Mean pre-pregnancy BMI^5^, kg/m^2^ (SD)	24.3 (4.8)	24.2 (4.2)	24.4 (4.4)
Pre-pregnancy BMI^5^ groups, n (%)			
<18.5	7593 (1.9)	149 (1.6)	40 (1.6)
18.5–24	123 615 (30.5)	3031 (32.7)	808 (31.3)
25–29	43 937 (10.8)	1139 (12.3)	357 (13.8)
30–34	16 403 (4.0)	437 (4.7)	136 (5.3)
≥35	7668 (1.9)	80 (0.9)	31 (1.2)
Missing^4^	206 507 (50.9)	4432 (47.8)	1211 (46.8)
Mean maternal height, cm (SD)	167.1 (6.3)	167.8 (6.5)	167.8 (6.2)
Offspring male sex, n (%)	569 588 (51.5)	8009 (51.6)	1615 (52.2)
Chronic maternal morbidity^6^, n (%)	166 499 (15.0)	2379 (15.3)	541 (17.4)
Culture duration, n (%)			
2–3 days	–	4942 (31.9)	1301 (42.1)
5 days	–	147 (1.0)	148 (4.8)
Missing	–	10 421 (67.2)	1644 (53.2)
Number of embryos transferred, n (%)			
1	–	7061 (45.5)	1795 (58.0)
2	–	7657 (49.4)	1235 (39.9)
3	–	581 (3.8)	52 (1.7)
Missing	–	211 (1.4)	11 (0.4)
Fertilization method, n (%)			
IVF	–	9228 (59.5)	1848 (59.8)
ICSI	–	6191 (39.9)	1230 (39.8)
Missing	–	91 (0.6)	15 (0.5)

Fresh-ET, fresh embryo transfer; frozen-ET, frozen embryo transfer.

1 Smoker status was recorded from December 1998 in a subpopulation of 769 677 pregnancies.

2 Mothers who smoked in the beginning or at the end of pregnancy.

3 Pregnancies where the mother did not consent to registration of smoking status were treated as missing smoking status in the analyses.

4 Missing during the registration period, from December 1998 to December 2015.

5 BMI was recorded from 2007 in a subpopulation of 417 574 pregnancies.

6 All pregnancies among mothers who in any pregnancy during the study period were registered with chronic hypertension, heart condition, kidney disease, diabetes mellitus, thyroid condition, rheumatoid arthritis, asthma, epilepsy, or recurrent urinary tract infections in the Medical Birth Registry of Norway.

### Birth size

**Figure 2.**
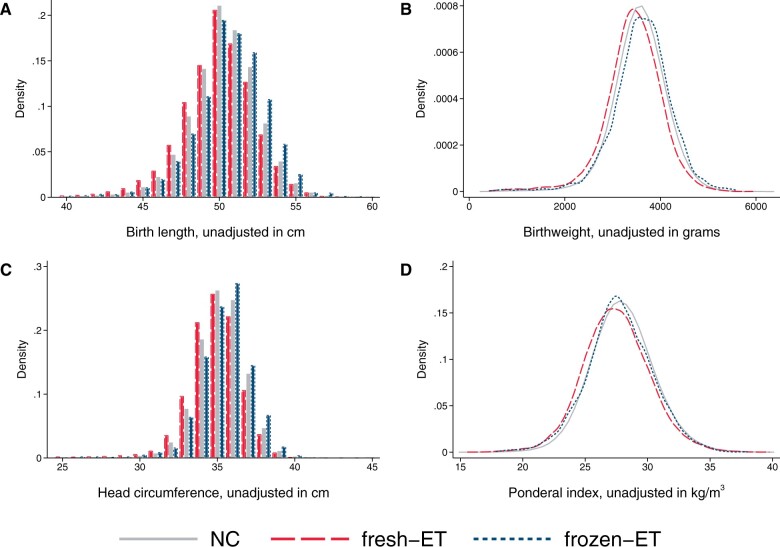
**Distributions of birth length (A), birthweight (B), head circumference at birth (C), and ponderal index at birth (D) according to mode of conception at the population level in the main analysis sample of liveborn singletons in Norway, 1988–2015.** Number of observations: NC n = 1 106 763, fresh-ET n = 15 510, frozen-ET n = 3093. NC, natural conception; fresh-ET, fresh embryo transfer; frozen-ET, frozen embryo transfer.

**Figure 3.**
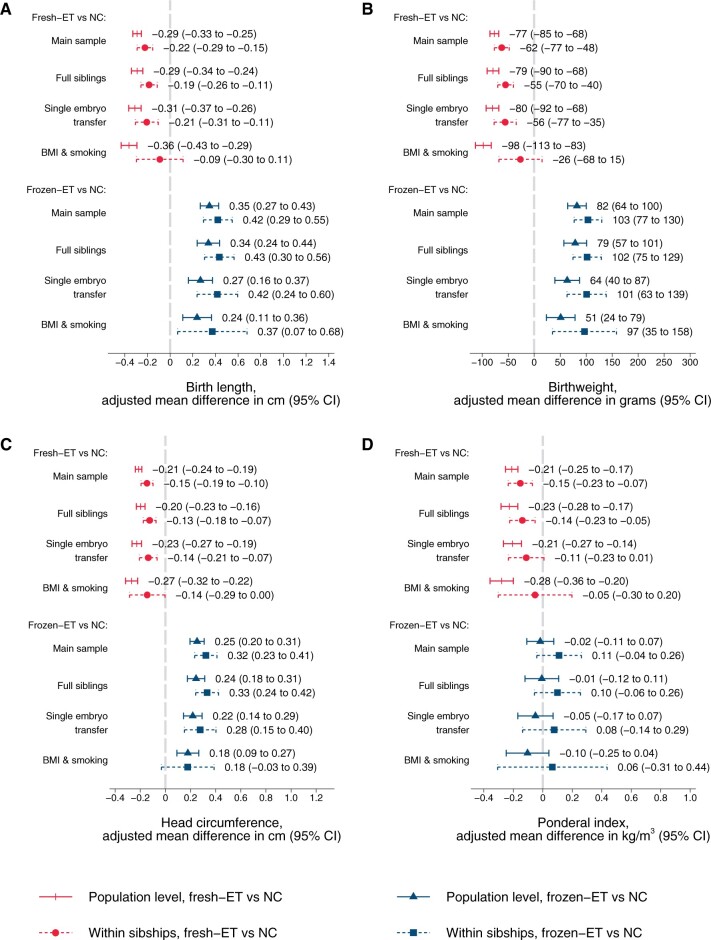
**Adjusted mean differences in birth length (A), birthweight (B), head circumference at birth (C), and ponderal index at birth (D) according to conception method for liveborn singletons in Norway 1988–2015: at the population level and within sibships.** Adjusted for year of birth, maternal age, parity, and education. Analyses in BMI and smoking sample include additional adjustment for maternal height, BMI, and smoking (maternal height is constant within sibships and therefore not included as a covariate in these analyses). NC, natural conception; fresh-ET, fresh embryo transfer; frozen-ET, frozen embryo transfer.

At the population level, singletons after frozen-ET had higher unadjusted mean values of birth length (50.5 cm), birthweight (3611 g), and head circumference at birth (35.4 cm) compared to singletons after NC (50.3 cm, 3571 g, and 35.2 cm, respectively, Table 2), but a similar ponderal index (frozen-ET: 27.8 kg/m^3^; NC: 28.0 kg/m^3^), reflecting shifts of population level distributions towards symmetrically larger sizes (Fig. 2). Unadjusted means in sibships were comparable to those at the population level (Table 2). The differences between frozen-ET and NC were confirmed and slightly stronger in the adjusted analyses, both at the population level and within sibships, and were broadly consistent across the different sensitivity analyses (Fig. 3).

**Table 2.** Unadjusted means and SDs of birth size, placental weight, and gestational age, according to mode of conception for liveborn singletons in Norway, 1988–2015.

	Population level, mean (SD)			Within sibships^1^, mean (SD)		
	**Natural conception n = 1** **106** **763**	**ART with fresh-ET n = 15** **510**	ART with frozen-ET n = 3093	Natural conception n = 6657	ART with fresh-ET n = 5871	ART with frozen-ET n = 1695
**Birth length, cm**	50.3 (2.4)	49.8 (2.8)	50.5 (2.7)	50.3 (2.5)	49.9 (2.7)	50.7 (2.6)
**Birthweight, g**	3571 (544)	3434 (594)	3611 (609)	3575 (566)	3468 (582)	3658 (578)
**Head circumference, cm**	35.2 (1.6)	34.9 (1.9)	35.4 (1.9)	35.2 (1.7)	35.0 (1.8)	35.5 (1.8)
**Ponderal index, kg/m^3^**	28.0 (2.6)	27.5 (2.7)	27.8 (2.7)	28.0 (2.7)	27.6 (2.6)	27.9 (2.6)
**Placental weight^2^, g**	675 (147)	675 (154)	702 (167)	680 (152)	680 (155)	711 (168)
**Birthweight:placental weight ratio^1^**	5.43 (0.94)	5.22 (0.95)	5.29 (0.97)	5.41 (0.96)	5.25 (0.95)	5.30 (0.97)
**Gestational age, days**	280.2 (12.2)	277.4 (14.5)	278.7 (14.7)	279.3 (12.8)	277.5 (13.7)	279.0 (13.6)
**Birthweight *z*-score^3^**	0.00 (1.17)	−0.18 (1.17)	0.20 (1.20)	0.06 (1.17)	−0.10 (1.16)	0.29 (1.16)

Fresh-ET, fresh embryo transfer; frozen-ET, frozen embryo transfer.

1 Sibships with at least two of the three different conception methods.

2 Number of observations in analyses of placental weight at the population level: NC n = 729 308, fresh-ET n = 13 317, frozen-ET n = 2926; within sibships: NC n = 5013, fresh-ET n = 4641, frozen-ET n = 1512.

3 Birthweight *z* scores are defined as standard deviations according to gestational age in days and sex using Marsal’s formulas.

At the population level, singletons after fresh-ET had lower unadjusted mean values of birth length (49.8 cm), birthweight (3434 g), and head circumference at birth (34.9 cm) compared to NC (Table 2), accompanied by a lower ponderal index (fresh-ET: 27.5 kg/m^3^), reflecting shifts of population level distributions towards lower values (Fig. 2). Unadjusted means in sibships were comparable to those at the population level (Table 2). Adjustment slightly attenuated the differences between fresh-ET and NC, but birth size and ponderal index remained smaller for children from fresh-ET at the population level and within sibships (Fig. 3). In sensitivity analyses with additional adjustment for BMI and smoking, estimates within sibships were closer to the null, although with wide CIs (Fig. 3).

In analyses with interaction terms between parity and combinations of conception method for each mothers’ first and second delivery, results for both frozen-ET and fresh-ET supported that the associations were not driven by specific subgroups (Supplementary Fig. S1). Accounting for gestational age and sex through *z*-scores of birth size gave results in the same direction as analyses based on direct measurements (Supplementary Fig. S2).

### Placental weight and birthweight:placental weight ratio

**Figure 4.**
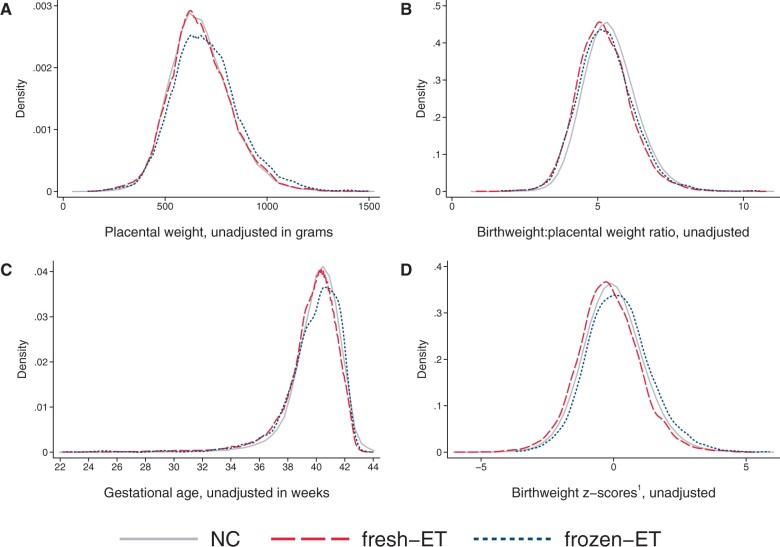
**Distributions of placental weight (A), birthweight:placental weight ratio (B), gestational age (C), and birthweight *z*-score (D) according to mode of conception at the population level in the main analysis sample of liveborn singletons in Norway, 1988–2015.** ^1^Birthweight *z*-scores are defined as standard deviations according to gestational age in days and sex using Marsal’s formulas (Marsal *et al.*, 1996). (**A**, **B**) Number of observations in analyses of placental weight: NC n = 729 308, fresh-ET n = 13 317, frozen-ET n = 2926. (**C**, **D**) Number of observations: NC n = 1 106 763, fresh-ET n = 15 510, frozen-ET n = 3093. NC, natural conception; fresh-ET, fresh embryo transfer; frozen-ET, frozen embryo transfer.

**Figure 5.**
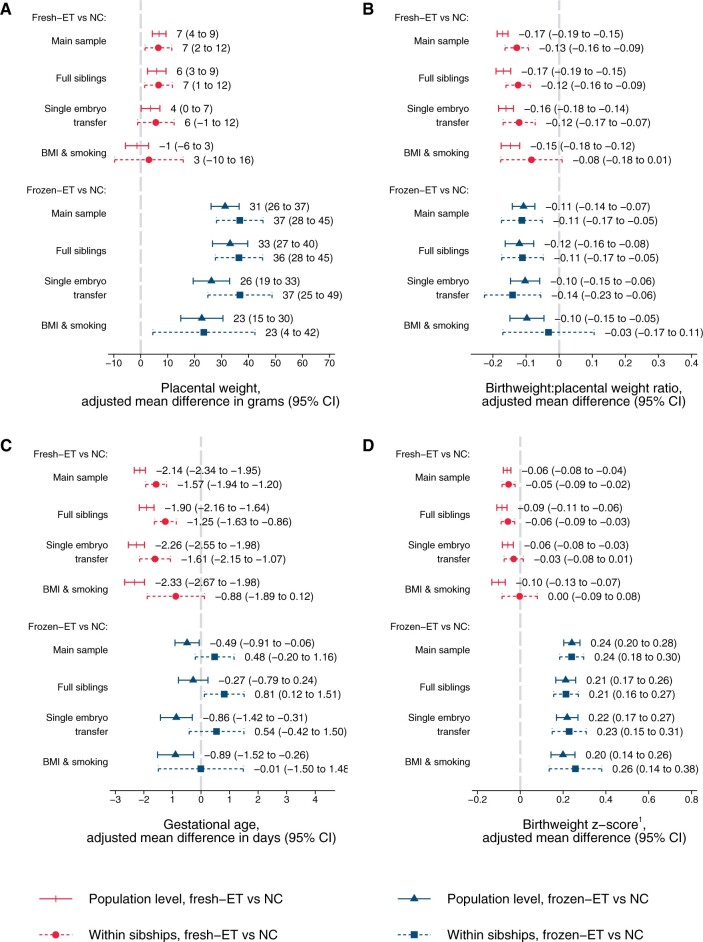
**Adjusted mean differences in placental weight (A), birthweight:placental weight ratio (B), gestational age (C), and birthweight *z*-score (D) according to conception method for liveborn singletons in Norway 1988–2015: at the population level and within sibships.** ^1^Birthweight *z*-scores are defined as standard deviations according to gestational age in days and sex using Marsal’s formulas (Marsal *et al.*, 1996). Adjusted for year of birth, maternal age, parity, and education. Analyses in BMI and smoking sample include additional adjustment for maternal height, BMI, and smoking (maternal height is constant within sibships and therefore not included as a covariate in these analyses). NC, natural conception; fresh-ET, fresh embryo transfer; frozen-ET, frozen embryo transfer.

At the population level, singletons after frozen-ET had higher unadjusted values of placental weight (702 g) compared to NC (675 g, Table 2), reflecting a shift of the population distribution towards higher values (Fig. 4A), whereas birthweight:placental weight ratio was slightly reduced (frozen-ET: 5.29; NC: 5.43) and shifted left towards lower values (Fig. 4B). These patterns of higher placental weight and lower birthweight:placental weight ratio persisted in adjusted analyses at the population level, and in unadjusted and adjusted estimates within sibships (Fig. 5A and B; Table 2). In sensitivity analyses with additional adjustment for BMI and smoking, differences within sibships in birthweight:placental weight attenuated, although with wide CIs (Fig. 5B).

At the population level, fresh-ET had similar unadjusted mean (675 g) and distribution of placental weight as NC (Fig. 4A), but birthweight:placental weight ratio was lower (5.22), and slightly shifted left (Fig. 4B). These associations were comparable to adjusted population level comparisons, and unadjusted and adjusted comparisons within sibships (Fig. 5A and B; Table 2). In sensitivity analyses with additional adjustment for BMI and smoking, differences within sibships in birthweight:placental weight attenuated slightly, although with wide confidence intervals (Fig. 5B).

The results for placental weight and birthweight:placental weight ratio were consistent in analyses with interaction term between parity and the different combinations of conception methods for each mothers’ first and second delivery, which indicate that the associations were not driven by specific subgroups (Supplementary Fig. S3A and B). Accounting for gestational age and sex through *z*-scores of placental weight and birthweight:placental weight ratio gave results in the same direction as analyses based on direct measurements (Supplementary Fig. S4A and B).

### Gestational age

In frozen-ET, the population level distribution of gestational age was wider than in NCs (Fig. 4C), and the mean gestational age was slightly lower in adjusted analyses at the population level, but attenuated completely within sibships (Fig. 5C). Fresh-ET had a similar population level distribution of gestational age as NCs (Fig. 4C), but a lower mean in adjusted analyses at the population level (Fig. 5C). Within sibships, the differences attenuated slightly, but still indicated lower mean gestational age (Fig. 5C).

## Discussion

In this large population-based study, children born after frozen-ET had a symmetrically increased birth size and higher placental weight than children born after NC, also when controlling for maternal factors through sibship analyses. In contrast, those born after fresh-ET had smaller birth size and were thinner at birth compared to those from NC. For both frozen-ET and fresh-ET, the birthweight:placental weight ratio was reduced compared to NC.

The main strengths of our study are the population-based design, and the ability to control for unobserved, constant maternal confounders through sibship analyses. An additional strength is the high data quality and rich information in the MBRN, which made it possible to explore differences in measures of birth size other than birthweight, while adjusting for multiple confounders. Prenatal care and reporting to MBRN are independent of conception method and women with ART pregnancies follow the same publicly financed prenatal care programmes as the background population. Thus, it seems unlikely that our findings may be the result of differences in follow-up or reporting. Reimbursement of costs related to fertility treatment in Norway ensures that the decision to choose ART treatment is based on medical indications rather than the couple’s private economy.

A limitation of our study is the smaller number of pregnancies with complete data on height, BMI and smoking, where we adjusted for highest number of potential confounders. In these analyses, results within sibships were very similar before and after additional adjustment for BMI and smoking (data not shown), which indicate that sample variations might drive inconsistencies with main findings rather than confounding. On the other hand, sensitivity analyses of full siblings were in line with main results, which control for constant parental factors. Although we cannot exclude confounding from non-constant factors, such as causes and duration of infertility, and parental changes in BMI, the consistency of results across order of conception method suggests that influence from such factors is limited. Furthermore, we had no data on treatment details, such as stimulation regimens, number of oocytes retrieved, embryo quality, culture medium, type of cryopreservation (i.e. slow-freeze or vitrification), or details on protocol for endometrial preparation in frozen-ET cycles (natural, stimulated, or programmed).

Pregnancies conceived in ART clinics outside Norway are only registered as ART-treatment if the mother discloses this to a midwife or doctor. Otherwise, they will be misclassified as a NC. However, this is unlikely to impact our results since they constitute a very small number compared to the large population of true naturally conceived pregnancies.

Birth length measurement is delayed or not performed in deliveries with breech presentation, and the incidence of breech presentation is higher after ART (Romundstad *et al.*, 2009). Although we cannot exclude a small selection bias for birth length, the proportion with missing birth length was low, and inclusion of pregnancies with missing birth length gave similar results on placental weight, head circumference, birthweight, and gestational age across conception methods (data not shown).

Correct birth length measurement depends on straight positioning and relaxed muscle tonus of the newborn, as well as type of equipment used for measuring (measuring board versus measuring tape) (Wood *et al.*, 2013). Similarly, placental weight depends on several factors such as trimming of membranes and umbilical cord, delayed umbilical cord clamping, and delayed placental weighing (Linderkamp *et al.*, 1992; Leary *et al.*, 2003). Therefore, measurement error of birth length and placental weight might differ slightly within and between delivery units. However, we have no reason to believe that it differs according to the type of conception method. The resulting non-differential misclassification would therefore lead to conservative estimates of the differences between the conception groups.

Chronic maternal morbidities, such as hypertension and diabetes mellitus, might influence growth and therefore confound our results. However, sibship analyses are less prone to such bias since each mother acts as her own control. Furthermore, exclusion of pregnancies by mothers registered with a chronic disease did not alter results (data not shown).

Within-sibship analyses require at least two deliveries by each mother, and comparison with NC requires ability to conceive naturally. These women might be less infertile than women who conceive after ART in general. Despite comparable birth and placental size measures between the sibship sample and the full population, some caution is therefore needed in generalization to all ART pregnancies.

The data used in this study are included in a recent Nordic sibship study of birthweight and gestational age (Westvik-Johari *et al.*, 2021), and our findings for these measures are, as expected, consistent with those results, also after adjusting for maternal education which was not possible in that study. The present study further details those findings by using a wider range of birth size and placental measures.

In line with our results, a Finnish study showed that unadjusted ponderal index was lowest in fresh-ET, highest in NC, and intermediate in frozen-ET (Pelkonen *et al.*, 2010). Further, Hann *et al.* (2018) also found that children born after frozen-ET had longer head circumference, whereas children born after fresh-ET had shorter head circumference at birth when compared to naturally conceived children.

The underlying mechanisms of the large size at birth after cryopreservation are not well understood. In the general population, maternal diabetes mellitus is an important risk factor for macrosomia (Persson *et al.*, 2011). However, in diabetic pregnancies, macrosomia is more often asymmetrical than in the background population (Persson *et al.*, 2011). In contrast, we found that the larger growth in frozen-ET is symmetrical.

In a study of singleton deliveries in Norway between 1999 and 2008, ART-conception was associated with larger placentas overall and lower birthweight:placental weight ratio (Haavaldsen *et al.*, 2012). Our study adds to those results by indicating that especially frozen-ET had larger placentas compared to NC. In pregnancies after frozen-ET, upregulation of placental growth might be a compensatory mechanism for increased foetal growth. Indeed, Ginod *et al.* (2018) showed that foetuses from both fresh-ET and frozen-ET were larger during the first half of the pregnancy, but that the large size persisted throughout pregnancy only in frozen-ET pregnancies. In addition, we found reduced birthweight:placental ratio for both frozen-ET and fresh-ET. In frozen-ET, increased risk of LGA and HDP have been linked to programmed cycles, possibly due to a missing corpus luteum in these cycles (Ginstrom Ernstad *et al.*, 2019). It is possible that our results for frozen-ET are driven by this subgroup, but unfortunately, we had no data to explore this further. However, our data suggest a shift of the entire distribution of placental weight, which might indicate that the association is present across subpopulations. Nevertheless, further research is warranted to establish the effect of programmed cycles with frozen-ET on placental size and efficiency.

In conclusion, the increased birthweight observed after frozen-ET was associated with a similar ponderal index compared to NC, which indicates a symmetrical growth pattern. In contrast, the lower birthweight in fresh-ET was associated with a reduced ponderal index compared to NC (asymmetrical growth pattern). For both frozen-ET and fresh-ET, there was a reduced placental efficiency, as measured by birthweight:placental weight ratio compared to NCs.

## Supplementary Material

dead094_Supplementary_Figure_S1

dead094_Supplementary_Figure_S2

dead094_Supplementary_Figure_S3

dead094_Supplementary_Figure_S4

## Data Availability

The data of this study are available from the Medical Birth Registry of Norway and Statistics Norway, but restrictions apply to the availability. These data were used under license for the current study and are not publicly available.
