# Supplementary material for: Birth size after embryo cryopreservation: larger by all measures?
Source: Hum Reprod. 2023 May 13;38(7):1379–89. doi: 10.1093/humrep/dead094 (PMC10320486; doi:10.1093/humrep/dead094)
Supplement: dead094_Supplementary_Figure_S1 [file dead094_supplementary_figure_s1.pdf]

OXFORD
human reproduction
Human Reproduction, 2023, 1–1
https://doi.org/10.1093/humrep/dead094
Supplementary Data

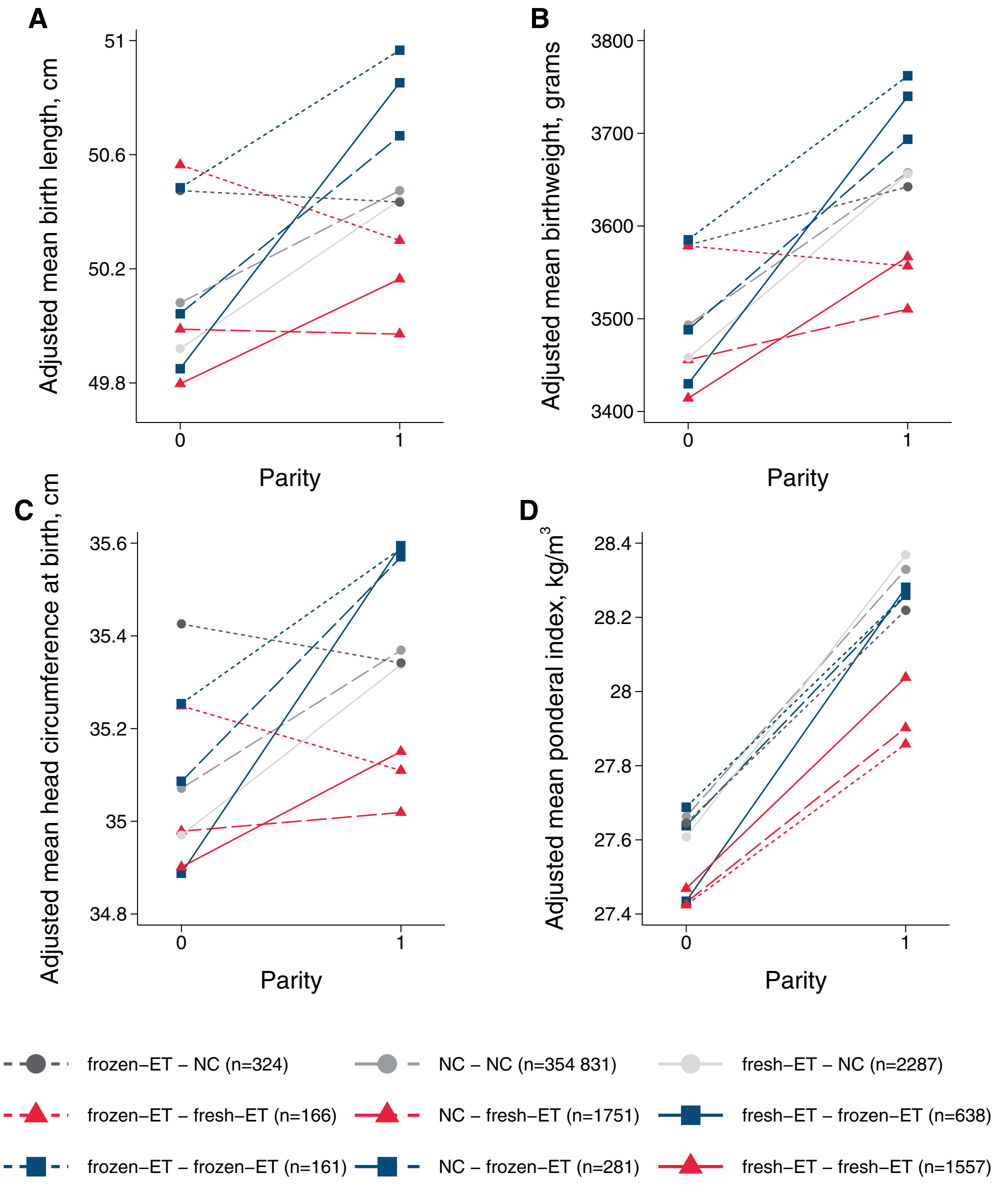

Supplementary Figure S1. Adjusted means of birth length (A), birthweight (B), head circumference at birth (C), and ponderal index at birth (D) in consecutive liveborn singletons sibling pairs according to parity and conception methods, born 1988–2015 in Norway. Means are estimated in mothers' first and second delivery in main sample using random effects linear models with post-estimation commands. Adjusted for birth year, maternal age, and education. NC, natural conception; fresh-ET, fresh embryo transfer; frozen-ET, frozen embryo transfer.
