# Supplementary material for: Birth size after embryo cryopreservation: larger by all measures?
Source: Hum Reprod. 2023 May 13;38(7):1379–89. doi: 10.1093/humrep/dead094 (PMC10320486; doi:10.1093/humrep/dead094)
Supplement: dead094_Supplementary_Figure_S2 [file dead094_supplementary_figure_s2.pdf]

OXFORD
human reproduction

Human Reproduction, 2023, 1–1
https://doi.org/10.1093/humrep/dead094
Supplementary Data

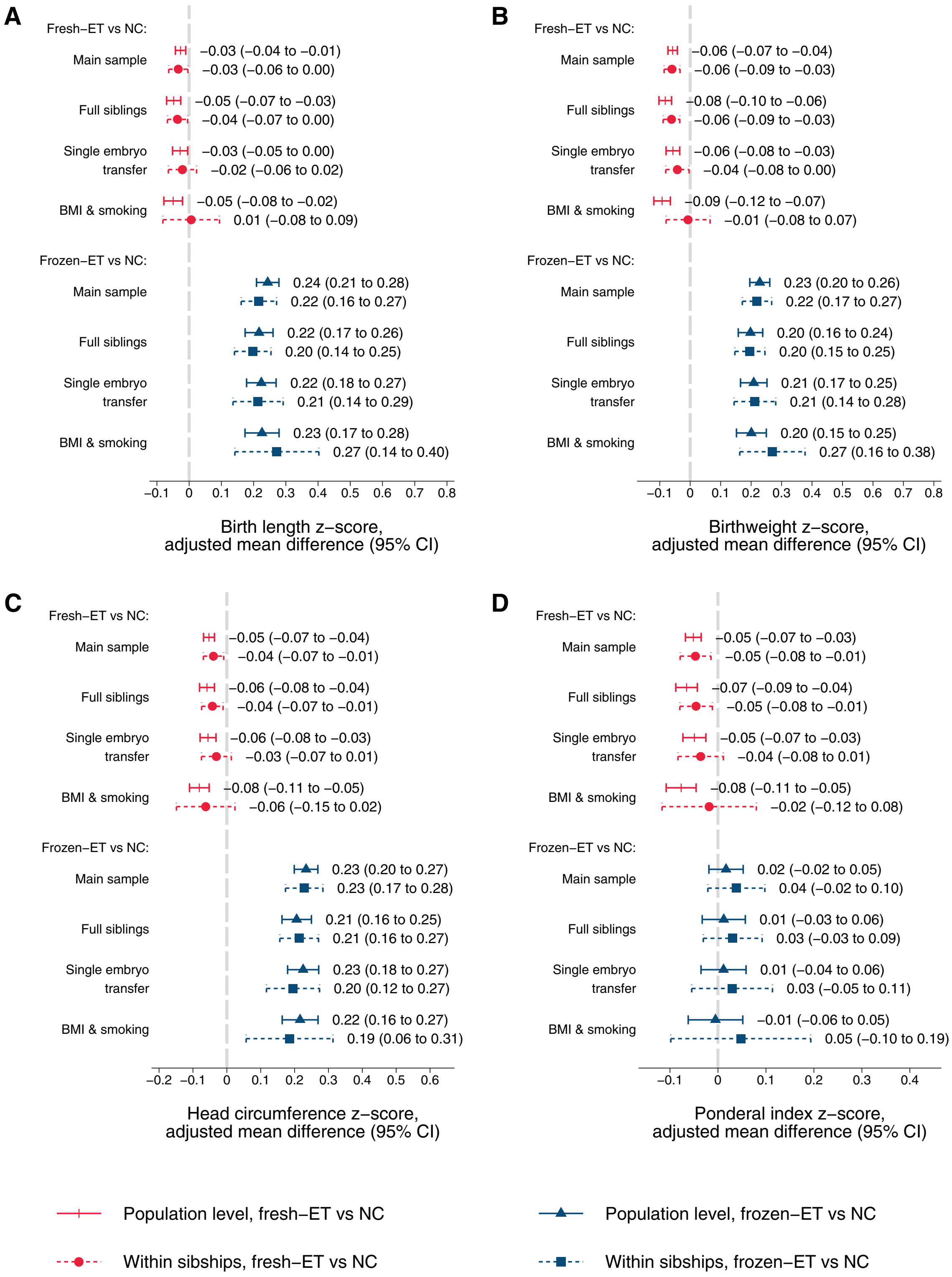

Supplementary Figure S2. Adjusted mean differences in z-score of birth length (A), birthweight (B), head circumference at birth (C), and ponderal index at birth (D) according to conception method in liveborn singletons 1988–2015 in Norway: at the population level and within sibships. All z-scores are estimated according to gestational age in days and sex using measured values at birth after NC as the standard. In comparison, birthweight z-score in Fig. 5 is calculated by using Marsal's formulas (Marsal *et al.*, 1996). Adjusted for year of birth, maternal age, parity, and education. Analyses in BMI and smoking sample include additional adjustment for maternal height, BMI, and smoking (maternal height is constant within sibships and therefore not included as a covariate in these analyses). NC, natural conception; fresh-ET, fresh embryo transfer; frozen-ET, frozen embryo transfer.
