# Supplementary material for: Birth size after embryo cryopreservation: larger by all measures?
Source: Hum Reprod. 2023 May 13;38(7):1379–89. doi: 10.1093/humrep/dead094 (PMC10320486; doi:10.1093/humrep/dead094)
Supplement: dead094_Supplementary_Figure_S3 [file dead094_supplementary_figure_s3.pdf]

OXFORD
human reproduction

Human Reproduction, 2023, 1–1
https://doi.org/10.1093/humrep/dead094
Supplementary Data

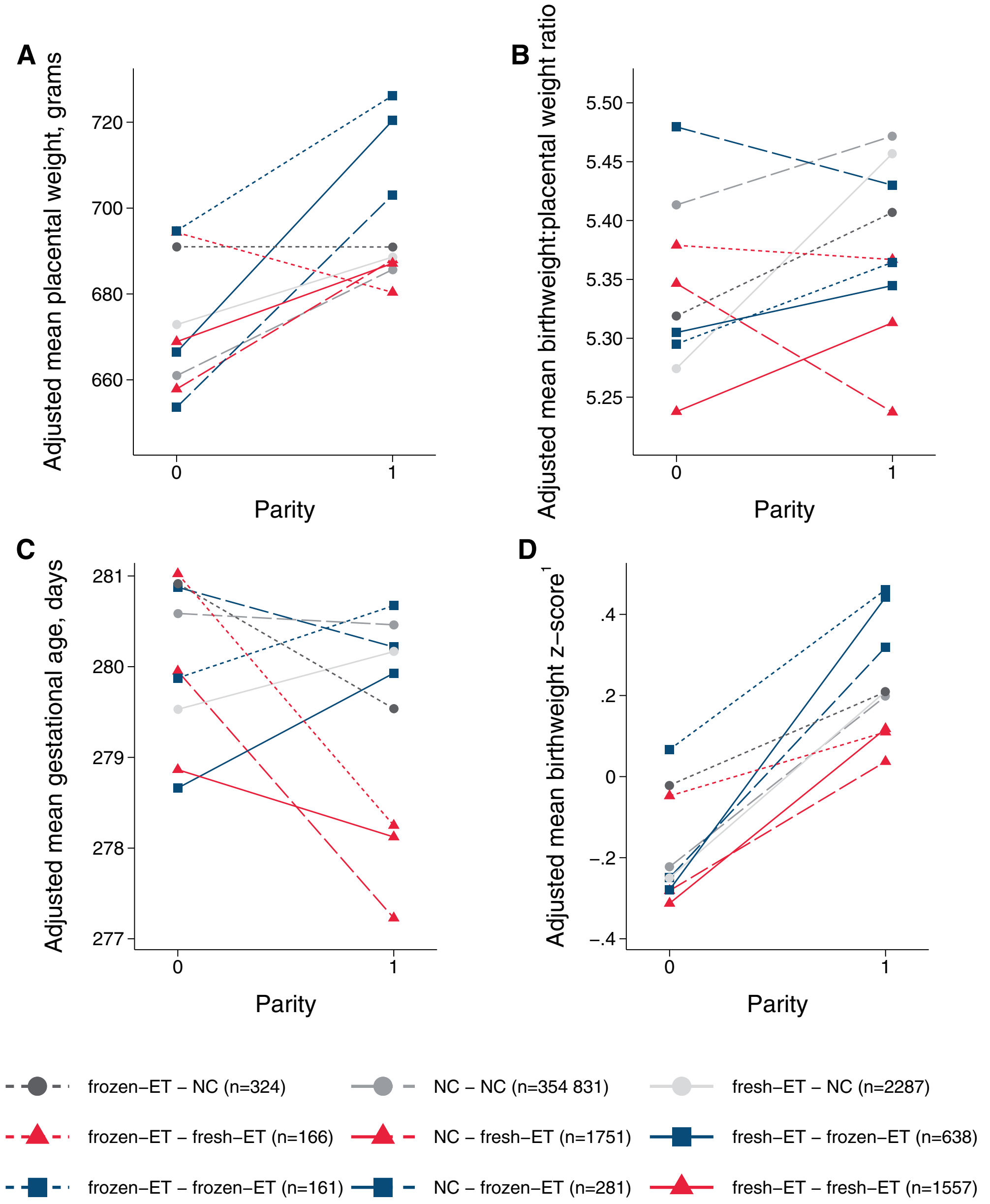

Supplementary Figure S3. Adjusted means of placental weight (A), birthweight:placental weight ratio (B), gestational age (C), and birthweight z-score (D) in consecutive singleton sibling pairs according to parity and conception methods, born 1988–2015 in Norway. [1]Birthweight z-scores are defined as standard deviations according to gestational age in days and sex using Marsal's formulas (Marsal *et al.*, 1996). Means are estimated in mothers' first and second delivery in main sample using random effects linear models with post-estimation commands. Adjusted for birth year, maternal age, and education. (A, B) Number of observations in analyses of placental weight: frozen-ET–NC n = 287, frozen-ET–fresh-ET n = 149, frozen-ET–frozen-ET n = 153, NC–NC n = 190 442, NC–fresh-ET n = 1107, NC–frozen-ET n = 203, fresh-ET–NC n = 1906, fresh-ET–fresh-ET n = 1292, fresh-ET–frozen-ET n = 596. NC, natural conception; fresh-ET, fresh embryo transfer; frozen-ET, frozen embryo transfer.
