# Supplementary material for: Birth size after embryo cryopreservation: larger by all measures?
Source: Hum Reprod. 2023 May 13;38(7):1379–89. doi: 10.1093/humrep/dead094 (PMC10320486; doi:10.1093/humrep/dead094)
Supplement: dead094_Supplementary_Figure_S4 [file dead094_supplementary_figure_s4.pdf]

OXFORD
human reproduction
*Human Reproduction*, 2023, 1–1
https://doi.org/10.1093/humrep/dead094
Supplementary Data

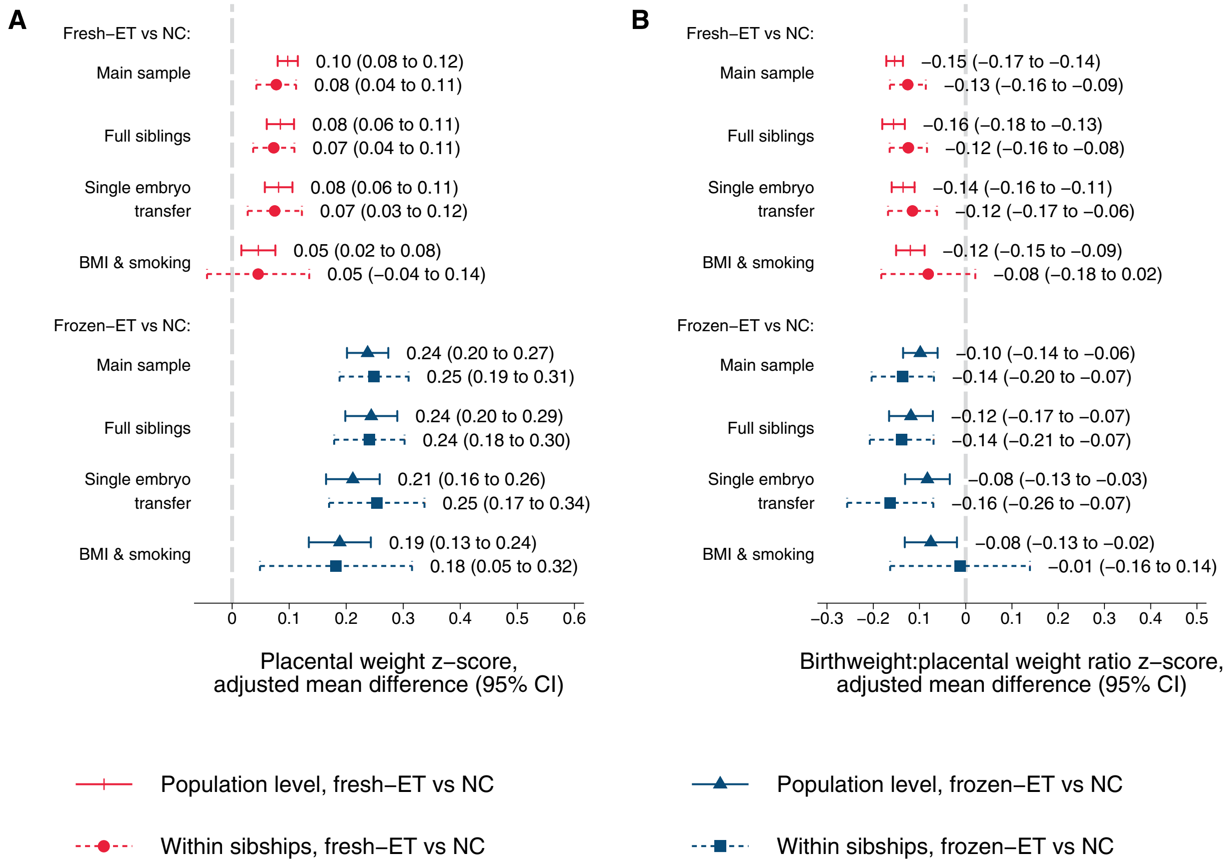


**Supplementary Figure S4. Adjusted mean differences in z-score of placental weight (A) and birthweight:placental weight ratio (B) according to conception method in liveborn singletons 1988–2015 in Norway: at the population level and within sibships.** All z-scores are estimated according to gestational age in days and sex using measured values at birth after natural conception as the standard. Adjusted for year of birth, maternal age, parity, and education. Analyses in BMI and smoking sample include additional adjustment for maternal height, BMI, and smoking (maternal height is constant within sibships and therefore not included as a covariate in these analyses). NC, natural conception; fresh-ET, fresh embryo transfer; frozen-ET, frozen embryo transfer.
